# Innovative upper extremity rehabilitation methods in stroke patients: a comparison of robotic rehabilitation and action observation therapy—a randomized trial

**DOI:** 10.3389/fneur.2026.1759263

**Published:** 2026-04-16

**Authors:** Emre Şenocak, Elif Korkut, Adem Aktürk, Aysel Yildiz Ozer

**Affiliations:** 1 Faculty of Health Sciences, Department of Physiotherapy and Rehabilitation, Karadeniz Technical University, Trabzon, Türkiye; 2 Department of Neurology, Bağcılar Education and Research Hospital, Istanbul, Türkiye; 3 Vocational School of Health, Department of Podology, İstanbul Gelişim University, Istanbul, Türkiye; 4 Department of Physiotherapy and Rehabilitation, Faculty of Health Sciences, Marmara University, Istanbul, Türkiye

**Keywords:** action observation therapy, motor function, quality of life, robotic rehabilitation, stroke

## Abstract

**Introduction:**

After a stroke, difficulties with arm and hand movement most affect daily life. To overcome these difficulties, research is ongoing to find the most effective treatment approaches. This study investigated which of two different technology-based rehabilitation approaches improved upper extremity motor function, independence, and quality of life in stroke.

**Methods:**

Thirty patients were randomized into two groups: robotic rehabilitation (RR) and action observation therapy (AOT). Upper extremity movements were repeated with active participation using the Houston Bionics ExoRehab X device in the RR group. On the other hand, the AOT group focused on watching and practicing functional movements through 31 first-person perspective videos. Motor functions, independence, and quality of life were assessed by the Wolf Motor Function Test (WMFT), Box-Block Test (BBT), Barthel Index (BI), and Stroke-Specific Quality of Life Questionnaire (SS-QoL).

**Results:**

The WMFT, BBT, BI, and SS-QoL scores of both groups improved from baseline. However, neither group was superior to the other in the first 4 weeks’ results. At the end of 8 weeks, the RR group demonstrated significantly greater improvements in all parameters compared with the AOT group (*p* < 0.05 for all) except SS-QoL (*p* = 0.136),

**Discussion:**

While both treatment methods showed similar short-term improvements, the RR group demonstrated greater improvements in motor function and independence than the AOT group at the end of 8 weeks. The fact that AOT gains slow down after the 4th week raises the question of which week these gains will stop.

**Clinical trial registration:**

Name: Effects of the Action Observation Therapy and Robotic Rehabilitation on the Upper-Limb Motor Function in Stroke. ID: NCT05590156 (18.04.2024). URL: https://register.clinicaltrials.gov/prs/beta/studies/S000CKXO00000049/recordSummary.

## 1 Introduction

Stroke may occur due to various pathophysiological mechanisms, including excitotoxicity, neuroinflammation, oxidative stress, and angiopathy, but ultimately results in neurological deficits (1). It most commonly affects movement, which can seriously impact daily activities and social participation (2). Motor deficits are frequently seen in the upper extremities, and upper extremity impairment is present in approximately 80% of patients in the acute phase and 40% of patients in the chronic phase (3). The rehabilitation process should be initiated immediately to resolve restrictions on activities and participation and achieve motor recovery. The first voluntary movement occurs 6–33 days after stroke, but these movements are usually compensatory (4, 5). Effective and efficient rehabilitation tools are needed to replace compensatory movements with voluntary motor movements.

Stroke rehabilitation aims to increase the patient’s autonomy by using all possible modalities to regain lost function (6). In addition to the direct effect of stroke, the use of inadequate and inappropriate rehabilitation approaches is also a determining factor in the low expected gains in the upper extremities (7). Many rehabilitative approaches are used to overcome motor recovery issues. Technology-based rehabilitation systems have also become increasingly common in stroke rehabilitation due to technological advances over the past few years (8).

A well-known category of such systems is computer-assisted robotic systems. Robotic systems enable intensified rehabilitation programs and task-oriented functional training to be applied repeatedly, thereby increasing motor recovery (9, 10). In addition, the strong feedback from the technological system increases patients’ motivation, potentially leading to better therapeutic outcomes.

Action Observation Therapy (AOT) is a multisensory (motor, somatosensory, and cognitive) rehabilitation strategy based on the principle that motor cortical areas are activated not only during the physical performance of actions but also during their mental practice or mere observation. In this method, patients are asked to observe a functional activity of a healthy role model on a screen for a while and to imagine performing the same movement. The neurophysiological basis of this mechanism is the Mirror Neuron System, formed by the rostral portion of the inferior parietal lobe, the pars opercularis of the inferior frontal gyrus, and the ventral premotor cortex (11). When a movement is observed, it has been shown that the motor representations in the brain that are active during its execution are also activated (12). The resulting internalized movement representation serves as a crucial target for motor and re-motor learning processes, laying the groundwork for neurological recovery (13). Mirror neurons support motor learning and facilitate movement execution by increasing motor system excitability through extensive cortical networks. Repeated observation and imitation of goal-oriented actions during the AOT process modulate the mirror neuron system, promoting the neuroplastic changes necessary for motor skill acquisition and improvement (14). In the context of neurological rehabilitation, this system is said to induce neuroplasticity by supporting the reorganization of partially damaged neural circuits and to offer an alternative neural pathway for the restoration of voluntary motor functions in individuals with motor deficits (11). The effectiveness of AOT is explained by the cortical and subcortical neural mechanisms underlying motor learning.

The neural basis of motor learning involves dynamic interactions between cortical and subcortical structures. Cortical regions, particularly the motor cortex, play a crucial role in the initial acquisition of motor skills and rapid adaptation to novel tasks (15). During AOT, observing a movement activates neural representations of that movement within the motor system. This process is explained by the direct matching hypothesis, which proposes that observed actions are matched with the observer’s internal motor representations. This matching process has been shown to involve regions such as the left inferior frontal cortex and the right superior parietal lobule (16). Consequently, even the mere observation of movement can elicit activation patterns in the motor system that resemble those during actual execution, thereby supporting the early stages of motor learning.

In the later stages of motor learning, subcortical structures play a more prominent role. The striatum integrates inputs from the motor cortex and the thalamus and plays a critical role in reinforcing learned motor behaviors. As motor skills become increasingly automated through repeated practice, reliance on cortical control decreases, while the contribution of subcortical systems becomes more prominent (17). Furthermore, the thalamus functions as a vital relay station, strengthening neural circuits through repetition (15, 17).

Recent neuroimaging studies further indicate that, beyond classical cortical mirror neuron system (MNS) regions such as the parietal, premotor, and prefrontal cortices, several subcortical structures—including the cerebellum, thalamus, and basal ganglia—also contribute to action observation processes. Errante and colleagues reported that both action execution and simple observation of hand grasping movements elicit activation across cortical, cerebellar, and subcortical regions, revealing a broadly somatotopic organization and shared neural recruitment across these structures (14). Although direct evidence for mirror neurons within subcortical nuclei remains limited, converging evidence suggests functional connectivity between the cerebellum, basal ganglia, and thalamus, as well as cortical MNS areas. Within this framework, the cerebellum has been proposed to play a supervisory role during action observation and imitation through feedforward and feedback control loops, contributing to predictive motor representations and minimizing sensorimotor prediction errors. Supporting this view, Abdelgabar et al. demonstrated that impairments in action perception correlate with the degree of cerebellar degeneration, highlighting the cerebellum’s importance in accurate action processing (18). Regarding the basal ganglia, regions such as the caudate nucleus, the medial sector of the motor putamen, and the globus pallidus have been implicated in both action observation and execution. At the same time, activation of the subthalamic nucleus has also been reported during the observation of hand movements (19). Given that the indirect pathway of the basal ganglia plays a critical role in inhibiting motor actions, the globus pallidus may help dissociate internally generated motor representations from overt motor output during action observation, thereby preventing unwanted motor execution (14).

Although the AOT has been increasingly studied in recent years, randomized controlled trials remain relatively few compared with more established rehabilitation techniques, such as robotic rehabilitation (11). Thus, this paper aimed to compare the impact of AOT and exoskeleton robotic rehabilitation systems, delivered in addition to conventional methods, on upper extremity motor function, independence, and quality-of-life parameters in patients with subacute stroke.

## 2 Methods

### 2.1 Ethics, randomization, and power

Marmara University Faculty of Medicine Clinical Research Ethics Committee approved this research (ID: 09.2022.649). The study was also registered in clinicaltrials.gov with the ID number NCT05590156.

An *a priori* sample size calculation was conducted using G*Power software (version 3.1.9.7) based on effect size estimates reported in previous studies by Lima and Christofoletti (20). Assuming the predefined alpha level and statistical power, the analysis indicated that a minimum total sample size of thirty stroke patients was required. Following sample size determination, participants were allocated using a simple randomization procedure. Random sequence generation was performed using an online randomization tool^1^. The predetermined sample size was entered into the software, and participants were assigned to the RR or AOT intervention arm in a 1:1 ratio. Each participant received the rehabilitation program corresponding to their allocated group. The non-blinded researcher conducted calculation.

Once the predetermined sample size (*n* = 30) was reached, the study’s *post-hoc* power analysis was also performed. For the effect size calculation, the Time*Group interaction partial eta-square of the Wolf Motor Function Test (η^2^ = 0.177) was used, and the effect size was determined as 0.463 based on this. An alpha error was set as 5%; effect size as 0.463; total sample size as 30; number of groups as 2; number of measurements as 3; correlation between measurements as 0.662; and epsilon value as 0.805. Using these data, the post-hoc power of this study was calculated to be 99%.

### 2.2 Participants

Inclusion criteria were as follows: Patients (1) between the ages of 18 and 80 who had one unilateral cerebrovascular event in the last six months, (2) Had Mini-Mental State Examination >24, (3) Patients who could sit independently without arm support for 2 min on chair, (4) Participants with the ability to actively initiate movement in all upper extremity joints, (5) Had Modified Ashworth Scale score ≤ 2 points for elbow, (6) Had Fugl Meyer Upper Extremity Motor Assessment Scale score between 20 and 60 points, (7) Had regular communication and cooperation skills.

Exclusion criteria were as follows: (1) presence of secondary neurological disease and orthopedic condition affecting the upper extremity, (2) receiving neuropsychiatric treatment, (3) had undergone Botox or relaxation surgery for the upper extremity, (4) using antiepileptic drug therapy, (5) had ataxia, (6) Attending less than 80% of the total number of sessions, and (7) had visual impairment that cannot corrected with glasses.

### 2.3 Interventions

All sessions were conducted under the supervision of an experienced physiotherapist. Before each session, participants were screened for contraindications. During the sessions, participants were continuously monitored for signs of discomfort, excessive fatigue, pain exacerbation, or any adverse reactions, verbally. However, no adverse events occurred during the study period. Participants who were unable to attend their scheduled session on the appointed day were included in a make-up session within the same week (within 5 days). Make-up sessions were conducted on a separate day.

#### 2.3.1 Common conventional rehabilitation protocol

The conventional rehabilitation program was planned individually according to the patient’s needs, functions, and expectations, including those of the upper and lower extremities. All groups received 60-min conventional rehabilitation programs 3 days a week for 8 weeks.

#### 2.3.2 Robotic rehabilitation (RR)

Robot-assisted rehabilitation was performed with the Houston Bionics ExoRehab X brand/model device. The system is stationary. It includes four different games (airplane game, mole shooting game, stone smashing game, and shopping game). The device allows movement in shoulder flexion-extension, abduction-adduction, elbow flexion-extension, supination-pronation, wrist flexion-extension, and ulnar-radial deviation. RR was performed for 60x3x8 minutes/day/week after the conventional treatment under supervision (21). No scheduled rest breaks were planned during the sessions. However, in the robotic rehabilitation group, participants were inherently at rest during task transitions while the system was being adjusted. Within a single session, participants completed three different games. The number of movement repetitions was not systematically tracked for RR.

Our system does not contain any motor that will create any push-pull force. It is completely controlled with the patient’s active participation, and all upper extremity movements can be trained in isolation. Before each session, the maximum range of motion limits was determined, and the patient was asked to perform the relevant tasks and movements within these limits repetitively. RR systems were calibrated to the maximum range of motion each patient could achieve at each joint before each session. The rehabilitation program progressed throughout treatment by adjusting the speed and resistance settings of the games in the system as needed. Speeds were gradually increased in subsequent sessions when patients demonstrated a stable range of motion control and adequate coordination. Higher speeds were not implemented unless movement accuracy was maintained.

#### 2.3.3 Action observation therapy (AOT)

Thirty-one videos based on upper extremity functions were created by researchers, such as grasping, reaching for objects, drinking water, folding napkins, etc., and all exercises were classified according to Brunnstrom Upper Extremity Motor Stages for AOT intervention. Each patient watched the videos appropriate to their Brunnstrom level from a pool of 31 videos. The videos for each function were limited to 3 min. The same exercise or component was shown to the patient in loops for each 3-min segment. The number of movement repetitions was not systematically tracked for AOT.

The patients sat in an isolated room, free from external disturbances, and watched all the videos recorded in first-person perspective on a 24-inch computer screen. First, a physiotherapist instructed the patient that observing the movement was essential rather than practicing, and after watching videos, the patient tried to perform the same movement. The AOT session was completed in ten cycles for simple movement. One cycle consisted of 3 min of observation and 3 min of practicing the same movement. Complex movements were broken down into components, and each component was observed and practiced separately. The treatment time was limited to 60 min for all movements. Patients performed all exercises under the supervision of a physiotherapist in the clinic for 60x3x8 minutes/day/week. AOT exercises are the same as in the previously published study (22).

### 2.4 Assessments

**Figure 1:**
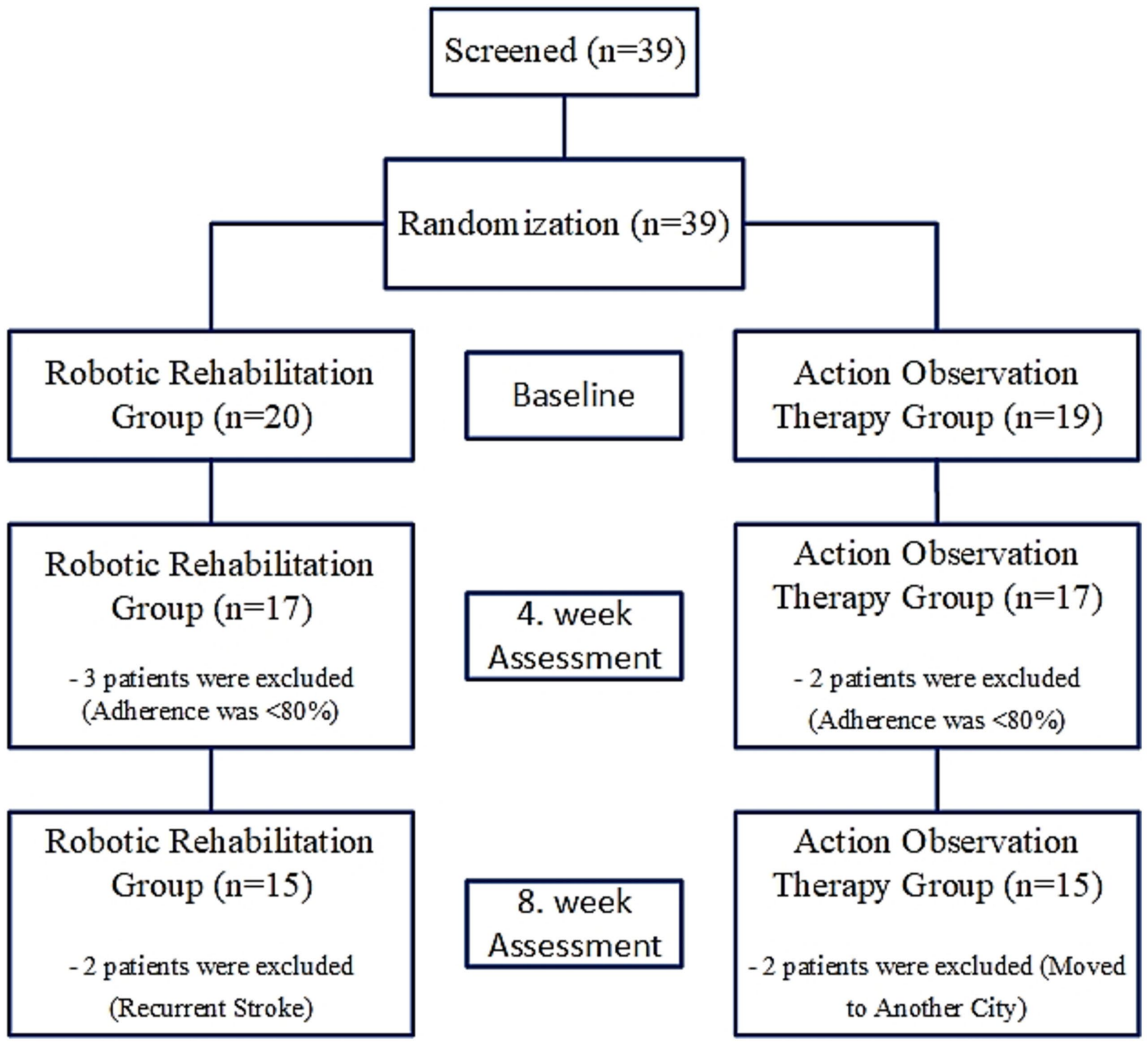
Flow diagram of the study.

Assessments were performed at three time points: baseline, 4th week, and 8th week (Figure 1).

#### 2.4.1 Demographic data form

This form contained age, body mass index (BMI), Fugl Meyer Upper Extremity Motor Assessment Scale score, gender, stroke onset time, lesion side, dominant extremity, stroke type, education level, and spasticity level.

#### 2.4.2 Fugl Meyer upper extremity motor assessment scale (FMA-UE)

This scale is a motor impairment scale specifically designed to assess recovery in hemiplegic patients after a stroke. It assesses all joint movements, coordination, and reflex activities related to the upper extremity. The total score ranges from 0 to 66, with a high score indicating good status (23).

#### 2.4.3 Wolf motor function test (WMFT)

The WMFT assesses the upper extremity’s motor skills and has 17 items. Fifteen of these items assess functional ability, and 2 assess strength. Since no hand dynamometer was available, the patient’s muscle strength could not be measured within the scope of this study. Each item, except muscle strength (two items), is scored on a 5-point Likert scale, with higher scores indicating greater functional ability (24). The maximum score for this study was 75.

#### 2.4.4 Box and block test (BBT)

The BBT is used to assess the manual dexterity of the hand. A wooden box with two compartments is used for the test. All cube blocks were placed in one compartment. The patient was asked to transfer the 2.5 cm cube blocks to the empty side as quickly as possible. It was emphasized that only one cube should be transferred each time. If more than one cube block was transferred, it was counted as a single block. This activity was repeated for 60 s, and the total number of blocks transferred was used as the score (25).

#### 2.4.5 Barthel index (BI)

Barthel index is a widely used scale to assess daily activities. The scale examines the unassisted performance of activities such as eating, going to the toilet, bathing, and going anywhere inside or outside the building. The scale’s total score is “100” points and indicates complete independence. A score of “0” indicates complete dependence (26).

#### 2.4.6 Stroke-specific quality of life (SS-QoL)

The SS-QoL, a disease-specific quality of life scale, comprises 12 domains (vision, language, thinking, personality, upper limb function, mobility, work and productivity, mood, energy, self-care, family roles, and social roles) and includes 49 items. Each domain has a maximum score of 5. The total score is 245, with a high score indicating a high quality of life (27).

### 2.5 Statistical analysis

Statistical Package for Social Sciences 11 software was used to analyze by non-blinded researchers. Participant numbers and percentages were used to define categorical variables, and mean ± standard deviation values were used for continuous variables. Pearson’s chi-square test was applied for categorical variables.

An independent samples t-test was used to compare just baseline assessments. Furthermore, a two-way mixed-model analysis of variance (ANOVA) was used to assess the impact of two interventions (RR and AOT) and time. Significant differences between measurements within a group were determined by Bonferroni correction. Partial eta-square was calculated as a measure of effect size. If a partial eta-square value of <0.06 represents a small effect, a value between 0.06 and 0.139 represents a medium effect, and a value >0.14 represents a large effect (28). Statistical significance was accepted as *p* < 0.05.

To control the effect of baseline score differences in the WMFT variable between the groups, a Repeated Measures Analysis of Covariance (Repeated Measures ANCOVA) was conducted. Pre-treatment WMFT scores were entered into the model as a covariate. The results were reported as baseline-adjusted estimated marginal means and standard errors.

There was no missing data, as all assessments were entered directly into the data analysis software at the time of evaluation. However, nine participants were excluded from the post-intervention analysis due to non-adherence to the study protocol (they had trouble accessing the hospital on their own), relocation to another city, or recurrent stroke. Although no missing data were observed, participant attrition occurred. Intention-to-treat analysis was not performed; instead, analyses were conducted using a per-protocol approach, including only participants who completed the study.

Even though we did not have missing data, we had drop-out, but we did not conduct an intent-to-treat analysis. The analyses were conducted using the data of all patients who completed the study.

## 3 Results

In this study, conducted with 30 patients with no adverse events, the RR group’s mean age was 55.40 ± 16.42 years, while the AOT group’s mean age was 50.66 ± 14.86 years (*p* = 0.415). The time of disease onset was also similar between the groups (*p* = 0.220). The results of motor impairment level assessed by FMA-UE and its score were 31.06 ± 5.11 points in the RR group and 35.66 ± 10.95 points in the AOT group (*p* = 0.152).

Both groups included more male than female patients (*N*_RR_ = 9; *N*_AOT_ = 10). Eighty percent of the patients in the RR group and 66.70% in the AOT group had a history of ischemic stroke. Data on all demographic characteristics of the groups are shown in Table 1.

**Table 1:** Characteristics of the patients.

Parameters	Sub-Groups	RR	AOT	p
Age (years)	–	55.40 ± 16.42	50.66 ± 14.86	0.415
Body mass index (kg/m^2^)	–	28.77 ± 5.78	26.36 ± 3.84	0.189
Stroke onset (month)	–	2.33 ± 1.17	3.00 ± 1.69	0.220
FMA-UE	–	31.06 ± 5.11	35.66 ± 10.95	0.152
Gender *N* (%)	Female	6 (40.00)	5 (33.33)	0.705*
	Male	9 (60.00)	10 (66.70)	
Education status *N* (%)	Primary School	12 (80.00)	8 (53.30)	0.329**
	High School	1 (6.70)	4 (26.70)	
	Bachelor’s degree	2 (13.30)	3 (20.00)	
Lesion side *N* (%)	Right	4 (26.70)	6 (40.00)	0.439*
	Left	11 (73.30)	9 (60.00)	
Dominant side *N* (%)	Right	14 (93.30)	13 (86.70)	0.543*
	Left	1 (6.70)	2 (13.30)	
Type of stroke *N* (%)	Ischemic	12 (80.00)	10 (66.70)	0.409*
	Hemorrhagic	3 (20.00)	5 (33.30)	
MAS *N* (%)	Score 0	7 (46.70)	6 (40.00)	0.532**
	Score 1	5 (33.30)	3 (20.00)	
	Score 1+	3 (20.00)	6 (40.00)	
Brunnstrom stage *N* (%)	4a	8 (53.30)	7 (46.70)	0.791**
	4b	3 (20.00)	2 (13.30)	
	4c	4 (26.70)	6 (40.00)	

*: Pearson Chi-squared Test. **: Fisher’s Exact Test; AOT: Action Observation Therapy Group; FMA: Fugl Meyer Upper Extremity Motor Assessment Scale; MAS: Modified Ashworth Scale; RR: Robotic Rehabilitation Group.

Data related to repeated measurements at different times are presented in Table 2. The baseline BBT (*p* = 0.679), BI (*p* = 0.133), and SS-QoL (*p* = 0.400) scores were similar. The WMFT score was 19.80 ± 14.52 points in the RR group and 34.66 ± 15.22 points in the AOT group. The baseline WMFT score was significantly higher in favor of the AOT group (*p* = 0.011). To robustly account for baseline imbalances, a Repeated Measures ANCOVA was conducted on the WMFT scores, with the baseline score entered as a covariate. The analysis yielded a statistically significant main effect of the intervention group (*p* = 0.031). Furthermore, a significant Time × Group interaction was observed (*p* = 0.015), indicating distinct trajectories of motor recovery between the two interventions. Specifically, the baseline-adjusted estimated marginal means indicated that, while both groups had similar adjusted scores immediately post-treatment (RR: 40.98 ± 3.44; AOT: 41.68 ± 3.44), the RR group showed a much larger improvement at the follow-up assessment (59.42 ± 4.33) than the AOT group (48.57 ± 4.33).

**Table 2:** Means, standard deviations of the all outcomes and it’s comparison of baseline values between groups.

Variables	Group	Time		
		*T*_0_	*T*_4_	*T*_8_
WMFT	RR	19.80 ± 14.52	35.33 ± 14.41	54.60 ± 15.35
	AOT	34.66 ± 15.22	47.33 ± 18.74	53.40 ± 20.75
	*p* ^a^	0.011**	NA	NA
BBT	RR	8.26 ± 8.47	20.60 ± 13.07	26.86 ± 15.16
	AOT	7.06 ± 7.17	12.46 ± 11.02	15.46 ± 11.72
	*p* ^a^	0.679	NA	NA
BI	RR	45.00 ± 24.56	74.00 ± 22.21	89.00 ± 22.69
	AOT	58.33 ± 22.57	76.66 ± 18.58	83.00 ± 18.00
	*p* ^a^	0.133	NA	NA
SS-QoL	RR	110.86 ± 37.71	145.80 ± 36.16	190.06 ± 34.79
	AOT	124.46 ± 48.74	158.33 ± 42.81	184.20 ± 31.78
	*p* ^a^	0.400	NA	NA

**AOT: Action Observation Group; BBT: Box and Block Test; BI: Barthel Index; FMA: Fugl Meyer Upper Extremity Motor Assessment Scale; NA: Not Assessed; *p*^a^: Independent Sample *T*-test *p*-value; RR: Robotic Rehabilitation Group; SS-QoL: Stroke Specific-Quality of Life; T_0:_ Baseline_;_ T_4_: 4th week; T_8_: 8th week; WMFT: Wolf Motor Function Test.

The mixed model analysis of variance (ANOVA) was used to determine whether repeated measurements differed between groups. When the Time*Group interaction was investigated, it was found that the WMFT (*p* = 0.008), BBT (*p* = 0.025), and BI (*p* = 0.040) results differed across time intervals between the groups (Table 3).

**Table 3:** Time × Group interactions of dependent variables.

Interaction	Variables	*F*	*p*	Partial η^2^
Time * Group	WMFT	6.043	0.008	0.177
	BBT	4.958	0.025	0.150
	BI	3.992	0.040	0.125
	SS-QoL	1.991	0.146	0.066

BBT: Box and Block Test; BI: Barthel Index; FMA: Fugl Meyer Upper Extremity Motor Assessment Scale; SS-QoL: Stroke Specific-Quality of Life; Wolf Motor Function Test.

The Bonferroni correction was applied to adjust for multiple comparisons between groups. The results are shown in Table 4.

**Table 4:** Multiple comparison of different measurements between groups.

Variables	Group	Time	Δ_change_	*p* ^a^	*p* ^b^	Partial η^2^
			Mean ± SD			
WMFT	RR	T_0_-T_4_	−15.53 ± 11.84	<0.001	0.545	NC
	AOT		−12.66 ± 13.71	0.009		
	RR	T_0_-T_8_	−34.80 ± 14.83	<0.001	0.012	0.205
	AOT		−18.73 ± 17.73	0.003		
	RR	T_4_-T_8_	−19.26 ± 12.42	<0.001	0.002	0.288
	AOT		−6.06 ± 8.72	0.052		
BBT	RR	T_0_-T_4_	−12.33 ± 12.09	0.004	0.076	NC
	AOT		−5.40 ± 8.13	0.066		
	RR	T_0_-T_8_	−18.60 ± 13.11	<0.001	0.017	0.188
	AOT		−8.40 ± 8.27	0.004		
	RR	T_4_-T_8_	−6.26 ± 5.62	0.002	0.054	NC
	AOT		−3.00 ± 2.82	0.003		
BI	RR	T_0_-T_4_	−29.00 ± 19.78	<0.001	0.141	NC
	AOT		−18.33 ± 18.77	0.006		
	RR	T_0_-T_8_	−44.00 ± 23.31	<0.001	0.031	0.156
	AOT		−24.66 ± 23.18	0.003		
	RR	T_4_-T_8_	−15.00 ± 14.14	0.003	0.059	NC
	AOT		−6.33 ± 9.53	0.066		
SS-QoL	RR	T_0_-T_4_	−34.93 ± 22.95	<0.001	0.918	NC
	AOT		−33.86 ± 32.43	0.004		
	RR	T_0_-T_8_	−79.20 ± 30.27	<0.001	0.136	NC
	AOT		−59.73 ± 38.71	<0.001		
	RR	T_4_-T_8_	−44.26 ± 24.06	<0.001	0.069	NC
	AOT		−25.86 ± 29.01	0.012		

AOT: Action Observation Group; BBT: Box and Block Test; BI: Barthel Index; NC: Not Calculated; *p*^a^: Repeated Measures ANOVA with Post-hoc Bonferroni Correction (for Within-Group Evaluation); *p*^b^: Multiple Comparison-Contrast Analysis significance values (for Between Groups); RR: Robotic Rehabilitation Group; SD: Standard Deviation; SS-QoL: Stroke Specific-Quality of Life; T_0:_ Baseline_;_ T_4_: 4^th^ week; T_8_: 8th week; WMFT: Wolf Motor Function Test; Δ_Change:_ Amount of Change between Times.

**Figure 2:**
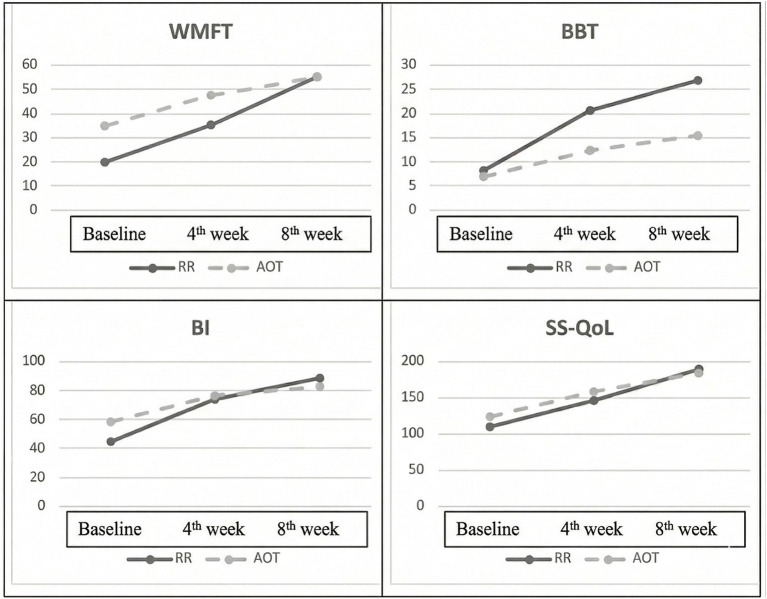
Graphs of variable changes over time.

All assessment parameters of the RR group differed from baseline to the 4th week (*p* < 0.05 for all). In the AOT group, WMFT (*p* = 0.009), BI (*p* = 0.006), and SS-QoL (*p* = 0.004) scores improved in the first 4 weeks. After the eight-week program, all assessments were statistically significantly different from baseline in both groups (*p* < 0.05 for all). In the assessments between the fourth and eighth weeks, the improvement rates of the groups varied. While the improvement levels of the RR group across all parameters increased between the 4th and 8th week (*p* < 0.05 for all), the recovery rates of the AOT group slowed (Figure 2). This slowdown occurred mainly in the WMFT (*p* = 0.052) and BI (*p* = 0.066) parameters.

According to inter-group analysis, the changes in WMFT, BBT, BI, and SS-QoL scores in the first 4 weeks were similar (*p* > 0.05 for all). The change in the first 8 weeks was greater in favor of the RR group in all variables (*p* < 0.05 for all) except the SS-QoL (*p* = 0.136). The change between the 4th and 8th weeks significantly differed in favor of the RR group in WMFT (*p* = 0.002). BBT, BI, and SS-QoL scores did not differ between the groups (*p* > 0.05 for all). The difference between the groups had a large effect size on all assessments (Partial η2 > 0.14 for all). The graphs of the variables’ changes are shown in Figure 2.

## 4 Discussion

The study primarily aimed to compare the effects of RR and AOT methods added to conventional exercise treatment on motor function in subacute stroke patients with upper extremity involvement. Secondary aims were to examine whether the methods applied would lead to changes in independence and quality of life. In this paper, improvement in motor function was assessed using the WMFT and BBT, and there was no difference between the groups during the first 4 weeks. However, at the end of the eight-week rehabilitation program, greater improvements were observed in the WMFT and BBT results in the RR group. After the next 4 weeks, the linear increase in the WMFT values of the RR group continued, while the improvement curve slowed down in the AOT group. BBT results showed a slowing trend in both groups after the first 4 weeks. Independence levels assessed by BI did not change between the groups in the first 4 weeks. Nonetheless, the RR group showed greater improvement over the first 8 weeks. There was no difference between the groups at any point, even though the RR group’s quality-of-life score was excellent across all assessment periods.

New-generation rehabilitation tools may help to achieve better rehabilitation gains (29). In our study, we compared the differences between two different technology-based adjunctive treatment methods. In a meta-analysis assessing motor function with the FMA, it was reported that RR and conventional methods yielded better outcomes than traditional rehabilitation alone (30). It is also known that exoskeleton robots provide better results in increasing arm functions (31). With this perspective, we used exoskeleton robotic rehabilitation in one group and AOT in the other, in addition to conventional methods.

It is possible to find studies examining the effects of robotic systems on motor function in stroke patients. A systematic review reported that RR yielded superior results compared to conventional methods in improving upper extremity motor function, especially in patients with chronic stroke, and emphasized that robotic systems may positively affect the recovery of arm function (31). The study conducted by Park et al. compared the effect of active-assisted and completely passive range of motion robots for people with a history of chronic stroke. As a result of the study, improvements in motor functions were reported in both groups (32). Another study comparing the effectiveness of manual hand rehabilitation intervention and robotic upper-extremity rehabilitation in chronic stroke found that the RR method did not demonstrate superiority in FMA scores. However, the proximal region of the upper extremity was better than that of the manual rehabilitation group. The probable reason for this study’s result is that the robotic system performs task-oriented movements related to the arm’s functionality rather than hand functions, whereas manual methods focus more on the hand (33).

Robotic rehabilitation enhanced finger movements in the early stage, improving hand motor functions, and yielded superior results in the RR group compared to the control group, as motor deficits peak in the first 3 months post-stroke (34). A few studies indicate that RR has a limited effect, whereas others suggest it is effective in improving motor function. The typical features of these studies were that the interventions were performed late in the course, the individuals had severe disability, and the robotic systems used were not specifically designed for the hand’s morphologic and anatomic structure (35, 36). Our study proved the positive effect of exoskeleton-type robotic rehabilitation, in addition to conventional methods, on upper extremity skills, motor function, and daily living activities in individuals with subacute stroke.

AOT is a new rehabilitation approach based on the principle of activating mirror neurons. Mancuso et al. examined the effects of AOT and task-oriented training on upper extremity function, and the improvement in the FMA score in the AOT group was greater than that in the other group, while the results for BBT were similar. It was emphasized that this study was conducted because patients observed the movements and imitated them, encouraging improvements in motor control that would allow them to reach maximum independence (37). According to our results, no difference was observed between the groups in WMFT and BBT in the first 4 weeks. We think that methodological differences, such as the number of videos watched per week, follow-up observation cycles, session duration, and inclusion criteria, were the basis of the difference between the results of Mancuso et al. and our paper.

Fu et al. also performed AOT in individuals with subacute stroke and sham AOT sessions consisting of geometric patterns, compared with a control group. As a result of the study, both FMA and WMFT test results were found to be higher in the AOT group. Authors emphasized that AOT increased mirror neuron activity and helped patients acquire new motor skills (38). In the study by Hsieh et al., a three-group study comparing AOT, mirror therapy, and bilateral arm training was planned, and it was reported that the FMA and BBT results of the AOT and bilateral arm training groups were similar. The functional videos shown to the AOT group were manually performed for the group receiving bilateral arm training. It was stated that the use of both AOT and bilateral arm training as treatment methods that require patient active participation may have affected the similarity of the results (39).

According to our results, the intra-group change caused by the RR intervention, especially during the first 8 weeks, had a large effect size for both upper extremity and hand motor functions. It is seen that passive and active-assistive robots are widely used in robotic systems, as reported in the literature. The device we used is a system that works entirely with the patient’s active participation and directly senses the patient’s movements. The device compensates for the patient’s limb weight, allowing the person to perform the movement directly. This facilitates patients’ ability to perform task-oriented activities by increasing the number of repetitions and difficulty levels. Although we did not record the number of repetitions in our study, the nature of the treatment programs differs. For example, the AOT has equal durations of observation and movement. This results in a 50% reduction in the time allocated to motor movement. On the other hand, the movement is directly attempted by the patient in the RR. The fact that the device supports the limb weight may have positively supported the number of movement repetitions. As a result, motor learning and neuroplasticity are thought to be triggered by increased synaptic co-activation and enhanced feedback from robotic systems. However, it should be noted that a significant difference was found between the groups in WMFT scores at baseline. Although the study used a randomized controlled design and there was no difference between groups in disease duration, this baseline disparity warrants caution in interpreting the results. The intervention effect was evaluated using a mixed-design ANOVA with a Group × Time interaction, an approach that accounts for change over time. Nevertheless, we consider the baseline difference a limitation of the study, given its randomization.

A 2021 systematic review found that AOT intervention is a treatment method with advantages for improving ICF sub-dimensions. However, due to the heterogeneity of the studies, no conclusions can be drawn regarding the treatment parameters (40). Another systematic review reported that AOT should be initiated as an additional treatment after the 23rd day after stroke; the sessions should last at least 30–40 min and should be applied for a minimum of 4 weeks (41). Our intervention period was more intensive and prolonged than recommended. The activity of mirror neurons, with the prolongation of time spent observing and imitating the action, may have improved motor-evoked potentials and motor learning skills.

In intergroup comparisons, the greater improvements observed in the RR group compared with AOT may be related to the motivational and performance feedback features of the system, the competitive nature of the games, and the possibility of performing three-dimensional movements, in addition to the functional characteristics of the RR device. The gross hand function (with BBT) result that RR did not differ from AOT in the changes in the first four and last 4 weeks is also essential. The probable reason for this may be that the device was not a robot for direct hand rehabilitation, which may have limited gains in arm function and the proximal upper extremity.

Patients’ performance skills in functional activities directly affect their independence in the post-stroke period. It has been observed that the positive effects of RR initiated in the early post-stroke period on functional independence are realized in the first 3 months (42). According to our paper’s results, no difference was observed between the groups in BI scores during the first 4 weeks. Over the eight-week period, greater improvements were observed in the RR group compared with the AOT group. Carpinella et al. investigated the effects of two rehabilitation approaches: an end-effector robotic device and a conventional therapy program. The authors reported that the RR group did not show superiority (43). In the study by Leem et al., improvements in the Functional Independence Measure (FIM) scores were reported among patients who underwent RR. The relationship between improvements in patients’ functional status and their level of independence enabled them to perform their activities of daily living more independently after robot-assisted therapy (44). Taravati et al. also applied both conventional treatment and RR as an adjunct to patients with chronic stroke. The study results showed increases in FIM scores for both the RR and the control groups, with group comparisons similar. This was attributed to the current robot’s inability to adequately train the hand’s fine motor skills and to a decrease in voluntary effort as the patient became accustomed to passive movements (45).

The number of AOT studies examining patients’ independence levels is small. In the study by Hsieh et al., only 1 of the 7 participants in the AOT group had an FIM change score exceeding the minimum clinically important difference. None of the participants in the other groups reached this threshold value (39). In another study, improvements in FIM scores were observed in both the AOT and control groups, with the difference between the groups reported to favor the AOT group. The increase in patients’ motor skills also explains changes in their independence levels (37). Nam et al. published an article, and while improvements were observed in the FIM scores of the AOT group and the control group, it was reported that there was no difference between the groups, and it was stated that no opinion could be expressed about the superiority of AOT intervention over conventional methods (46). According to our results, short-term treatment (4 weeks) did not affect independence levels between the groups, but the RR group showed better BI scores with prolonged treatment. In the AOT group, gains increased linearly in the first 4 weeks, but slowed after the fourth week. The possible reason for this may be that there are similarities in motor function gain results. Motor function gains of the RR group were higher than those of the AOT group. Therefore, we think the increase in motor gains may have led to the rise in functional skills.

As human life expectancy increases, assessing the quality of life in individuals with chronic diseases has become increasingly important (47). While 85% of stroke patients experience hemiparesis immediately after stroke, 55 to 75% of survivors continue to experience motor deficits, with a decrease in quality of life (48). According to our study, stroke patients’ quality-of-life scores were low in the pre-treatment period. Both treatment methods resulted in a similar increase in the patient’s quality of life. Many studies state that RR improves the quality of life in stroke patients (49, 50). The role of RR in improving quality of life is generally seen as resulting from improvements in hand function. Hsieh and colleagues also examined the effects of AOT on the quality of life in patients with subacute stroke. As a result, improvements in quality-of-life scores were observed in both the AOT group and the active control group. It was emphasized that the change observed in these two groups may be due to the similarity of the main change and the exercises applied to each group (39). In another study by Dettmers et al., home-based AOT was applied to patients with chronic stroke, and the patients’ quality of life improved (51).

Our study used a different method from many studies in the literature. For example, our most important differences were an eight-week treatment period, each AOT session lasting 60 min, and watching and practicing a video for three sessions instead of one functional video per session. Despite this, there was no difference between the groups in SS-QoL results, favoring neither RR nor AOT. However, an increase in the quality of life was observed in both groups. Impaired arm and hand function causes significant limitations in the performance of daily living activities, directly affecting quality of life (52). In intra-group evaluations, improvements in individuals’ quality of life can be explained by gains in upper extremity function. However, the partial increase in hand gains relative to other parameters in both groups may have led to similar results.

In conclusion, both groups showed improvements in WMFT, BBT, BI, and SS-QoL scores compared with baseline. In intergroup comparisons, greater improvements were observed in the RR group for all outcomes except quality of life. However, it should not be forgotten that the higher baseline WMFT scores in the AOT group indicate that these participants started closer to the ceiling for this measure, potentially limiting the magnitude of observable improvement. This ceiling effect should be considered when interpreting the greater improvements observed in the RR group at T8 for WMFT. For the other outcome measures, since baseline values were comparable, the observed between-group differences in improvement can be interpreted without adjustment for initial group differences. While the improvement trend in most assessment parameters of the RR group showed a linear increase after the fourth week, the recovery rate of all parameters of the AOT group slowed down after the fourth week.

This study is the first to compare the effects of robot-assisted rehabilitation systems and AOT interventions. Although we believe it will make a significant contribution to the literature in this respect, our research has some limitations. When the graphs in our analysis are examined, it is seen that the recovery curve tangent for the AOT treatment decreases over time. This situation has raised curiosity about whether the healing effect of AOT will plateau. The lack of long-term follow-up assessment limits our ability to comment on the duration of the AOT. Additionally, our patients were in the subacute phase. It is necessary to investigate whether the results obtained with AOT in earlier rehabilitation periods differ from those with RR. Also, 5 patients were excluded from the study after the first 4 weeks. The number of extracted patients was similar in both groups. Although this exclusion may seem negative, the fact that patients showed low participation could directly affect the study’s validity when sample size is taken into account. Another limitation was that the video pool in the AOT intervention had a limited number of videos. The ExoRehab X device used for RR is a complex upper extremity rehabilitation tool. It only allows wrist flexion-extension and ulnar-radial deviation, and grasping movements can also be performed for the hand. Since it is not a hand-specific robot system, the gains that can be achieved may be limited.

Also, one of the main limitations of the present study is that the participants were 2–3 months post-stroke, a period characterized by substantial spontaneous neurological recovery. As no conventional-therapy control group was included, it is not possible to fully disentangle the observed improvements from natural recovery processes. The reason for not including a control group was that, during the subacute period—which is critical for functional recovery—it would not have been ethically feasible to deprive patients of additional therapeutic intervention.

On the other hand, all participants had Mini-Mental State Examination (MMSE) scores≥24, indicating adequate global cognitive function to follow instructions. However, it is well recognized that the MMSE does not reliably assess visuospatial deficits, unilateral neglect, or apraxia. Furthermore, although cognitive eligibility was established using the MMSE, mild visuospatial or motor planning deficits cannot be completely ruled out. Therefore, the physicians’ baseline clinical evaluations documented that no visuospatial impairments or neglect were present in the patients.

Nevertheless, the lack of a more comprehensive cognitive assessment constitutes one of the limitations of our study. This may represent a limitation in terms of group homogeneity and the interpretability of treatment effects. Future studies are therefore recommended to include specific neuropsychological assessments to evaluate these functions. Additionally, the researchers who included the study were responsible for administering the treatment, performing outcome assessments, and calculating the sample size. The researchers were not blinded to group allocation. Due to the nature of the physiotherapy intervention, blinding was not feasible. This is acknowledged as a limitation of the study. Lastly, although *a priori* power analysis was conducted, the effect size used was based on the most methodologically similar study available, as no previous study used exactly the same methodology. Therefore, the estimated sample size may not perfectly reflect the true effect size in the current study, which should be considered a limitation. However, when a study is conducted on robotic rehabilitation and AOT, our study can be referenced, and a power analysis can be performed.

## Data Availability

The original contributions presented in the study are included in the article/supplementary material, further inquiries can be directed to the corresponding author/s.

## Footnotes

1
RandomReady.com
